# Supplementary material for: Human iPSC-Derived Retinal Organoids and Retinal Pigment Epithelium for Novel Intronic RPGR Variant Assessment for Therapy Suitability
Source: J Pers Med. 2022 Mar 21;12(3):502. doi: 10.3390/jpm12030502 (PMC8951517; doi:10.3390/jpm12030502)
Supplement: Supplementary file 1 [file jpm-12-00502-s001.zip › jpm-1589166-supplementary.pdf]

Supplementary Table S1. Primers for pluripotency characterisation of iPSC lines

**Target**	**Primer sequences (5' to 3')**
OCT4	Fwd: AGAAGCTGGAGCAAAACCCG;<br>Rev: TCCCAGGGTGATCCTCTTCT
NANOG	Fwd: CCTCCAGCAGATGCAAGAAC;<br>Rev: AAGGCTGGGGTAGGTAGGTG
SOX2	Fwd: ATGTCCCAGCACTACCAGAG;<br>Rev: GCACCCCTCCCATTTCCC

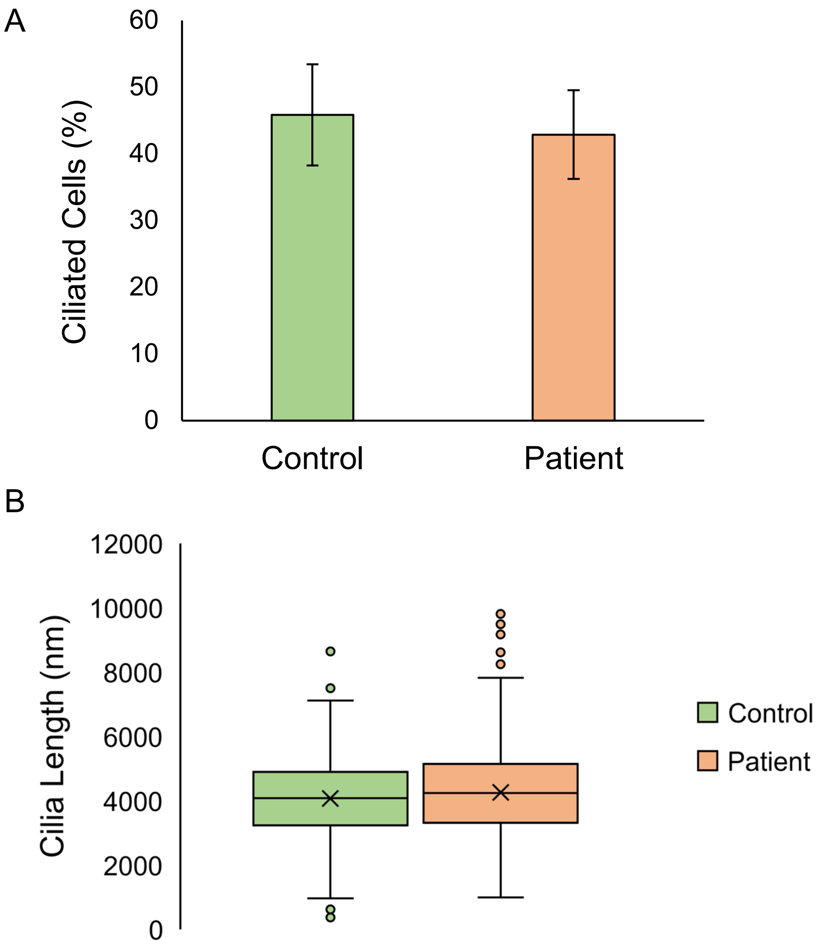

Supplementary Figure S1. Ciliogenesis and cilia length in fibroblast cells. *Fibroblast cilia were marked with acetylated α-tubulin.* ***A)*** *Ciliogenesis was calculated as a percentage of total number of cilia/total nuclei in the field of view. There were no significant differences seen in the percentage of ciliated cells between the control and patient groups (control cells counted n=764, patient cells counted n=663, p=ns).* ***B)*** *Cilia lengths of patient fibroblasts were measured and were found to be comparable to cilia lengths in control cells (unpaired t-test, P=ns, SEM from n=3 independent experiments, control cilia measured n=334, patient cilia measured n=260).*

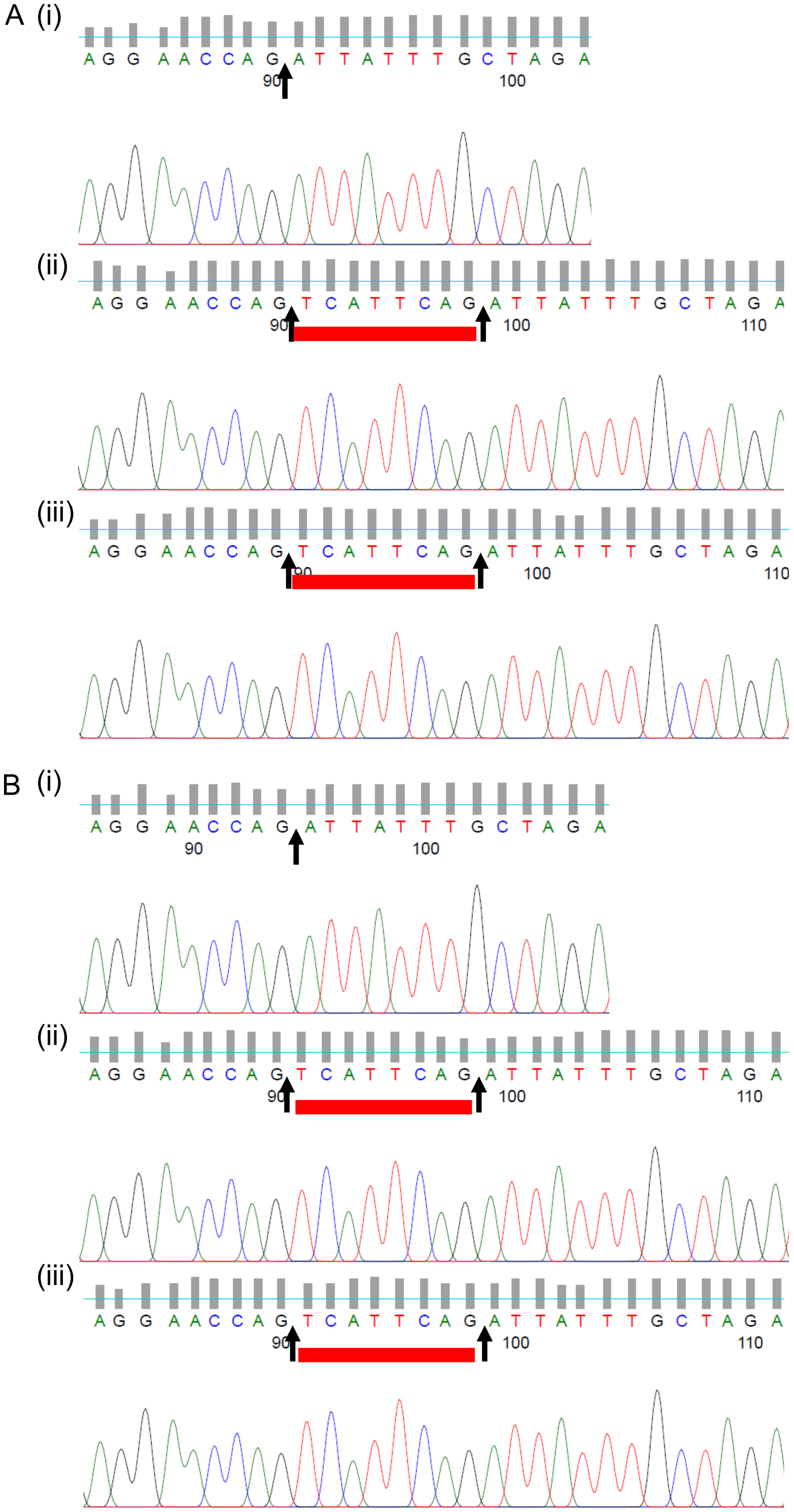

Supplementary Figure S2. Splice sequence confirmation in iPSC-RPE and iPSC-ROs. A) *Sequencing of amplified iPSC-RPE cDNA from **(i)** Control 1 showed canonical RNA splicing at the exon 11-12 junction (black arrow). In **(ii)** Patient Clone 1 and **(iii)** Patient Clone 2 iPSC-RPE, sequencing confirmed inclusion of the intronic sequence tcattcag (red underline) between the exon 11-12 junction (black arrows). **B)** Sequencing of amplified iPSC-RO cDNA from **(i)** Control 1 showed canonical RNA splicing at the exon 11-12 junction (black arrow). In **(ii)** Patient Clone 1 and **(iii)** Patient Clone 2 iPSC-RO, sequencing confirmed inclusion of the intronic sequence tcattcag (red underline) between the exon 11-12 junction (black arrows).*

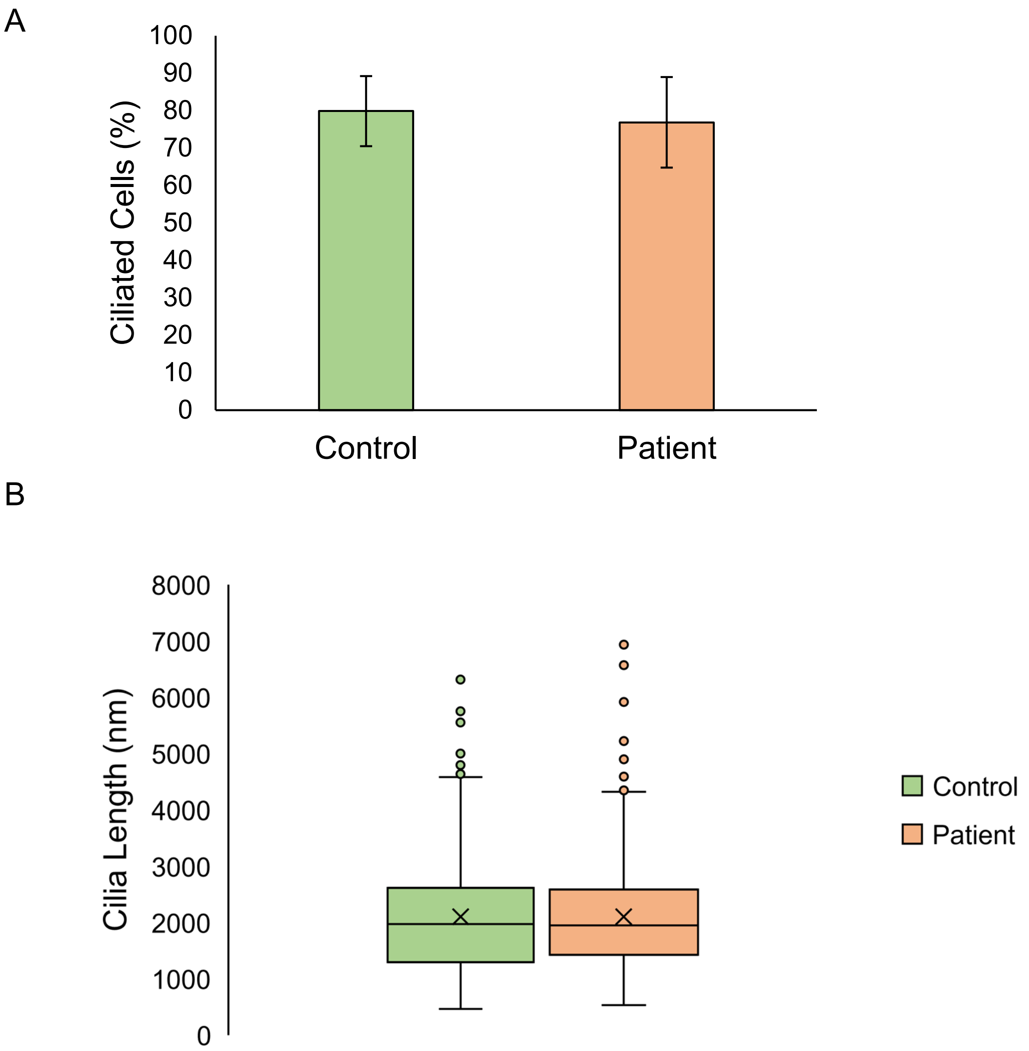

Supplementary Figure S3. Ciliogenesis and cilia length in iPSC-RPE cells. *iPSC-RPE cilia were marked with acetylated α-tubulin.* ***A)*** *Ciliogenesis was calculated as a percentage of total number of cilia/total nuclei in the field of view.* ***B)*** *Cilia length of patient iPSC-RPE was measured and were comparable to cilia lengths in control cells. There were no significant differences seen in the percentage of ciliated cells or in cilia length between the control and patient groups (unpaired t-test, P=ns, SEM from n=3 independent experiments, Control cilia counted n=474, Patient cilia counted n=412). (Control = Controls 1 and 2, Patient = Clones 1 and 2).*

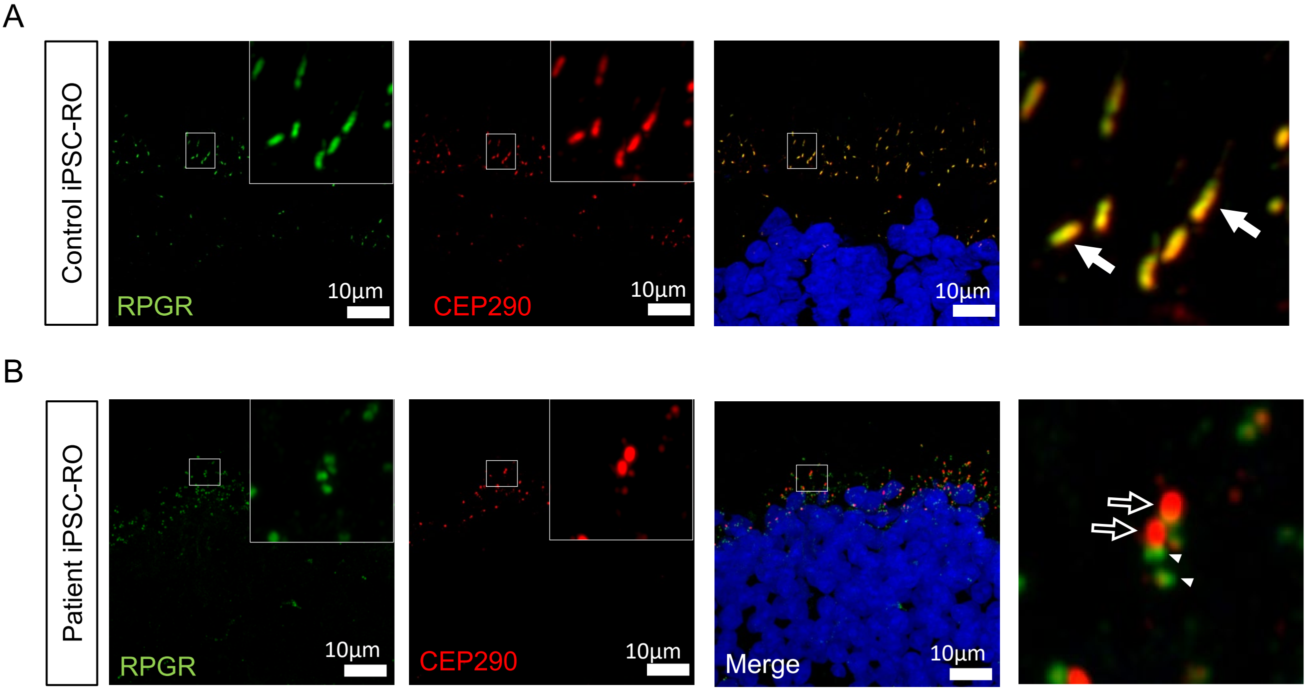

Supplementary Figure S4. Additional control and patient RPGR and CEP290 staining in iPSC-ROs. *Control iPSC-RO and patient iPSC-RO photoreceptor cells at age 30 weeks were stained with antibodies against RPGR (green) and CEP290 (red) which localizes mainly to the TZ of photoreceptor cilia.* ***A)*** *Control iPSC-ROs showed strong yellow co-staining (white arrows) along the ciliary TZ.* ***B)*** *Patient iPSC-ROs showed lack of RPGR staining and hence lack of co-staining with CEP290 at the photoreceptor TZ (white arrow outlines). There was presence of RPGR staining just away from the TZ region, which may be consistent with localization in the basal bodies (green punctate staining denoted by white arrowheads). (Control = Control 2, Patient = Clone 1).*

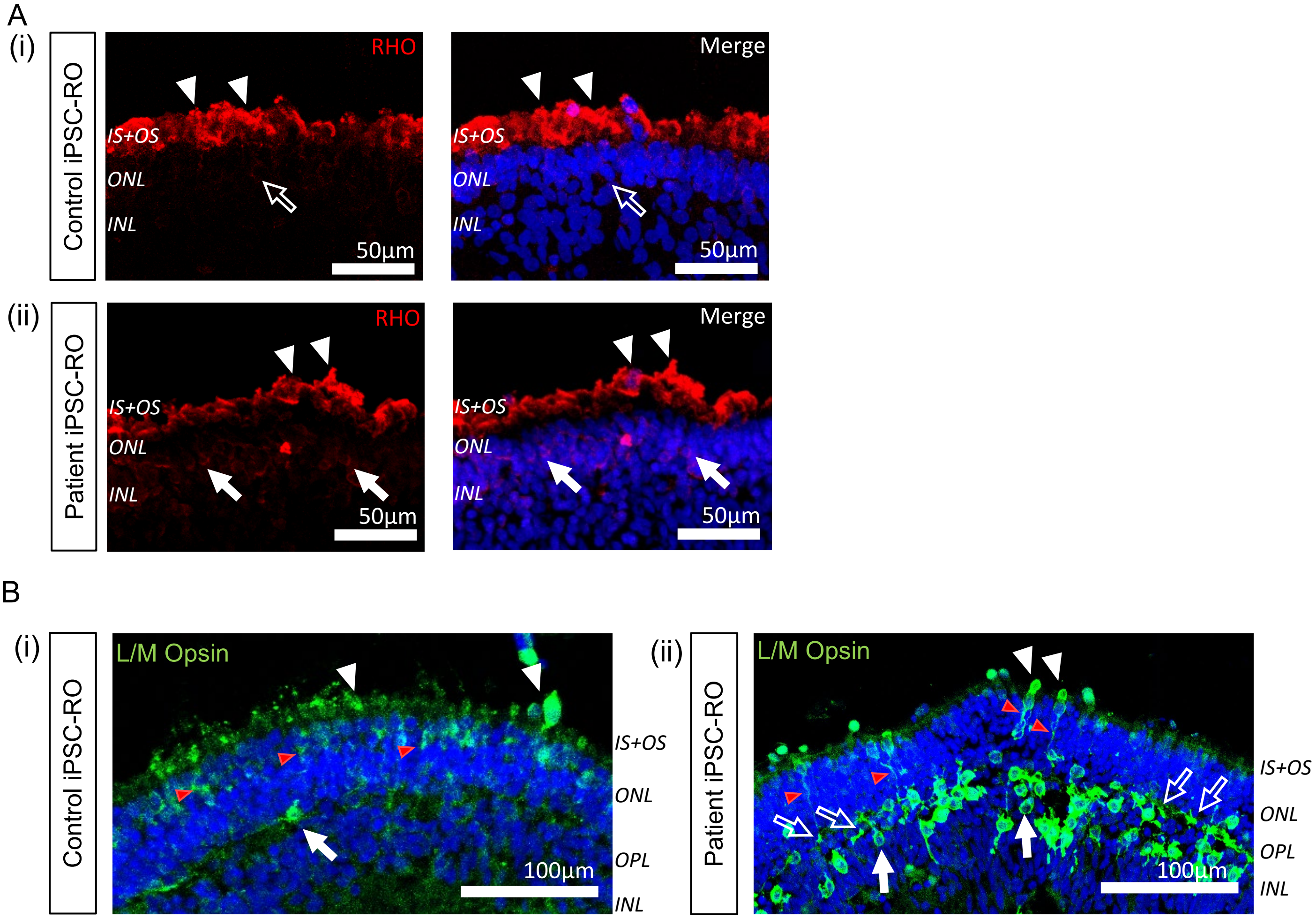

***Supplementary Figure S5. Additional control and patient rhodopsin and L/M opsin staining in iPSC-ROs. A) (i)** Rhodopsin staining (red) in control iPSC-ROs shows protein localization to the IS+OS region of rod photoreceptors (white arrowheads) with minimal staining in the cell soma (white arrow outline). **(ii)** In patient iPSC-ROs, staining is also seen in the IS+OS region (white arrowheads) and additional staining is present in the soma of the cells (white arrows). (Control = Control 1; Patient = Clone 2). **B) (i)** Expression of L/M opsin (green) was seen in the control iPSC-RO photoreceptor IS+OS region (white arrowheads) and ONL (red arrowheads), with some staining in the OPL and INL (white arrow). **(ii)** This pattern of staining was also seen in patient iPSC-RO (white arrowheads), with L/M opsin staining also seen in the OPL and INL (white arrows). Additional staining was seen in the OPL that was incongruent with L/M opsin soma staining (white arrow outlines). (Control = Control 1; Patient = Clone 1).*

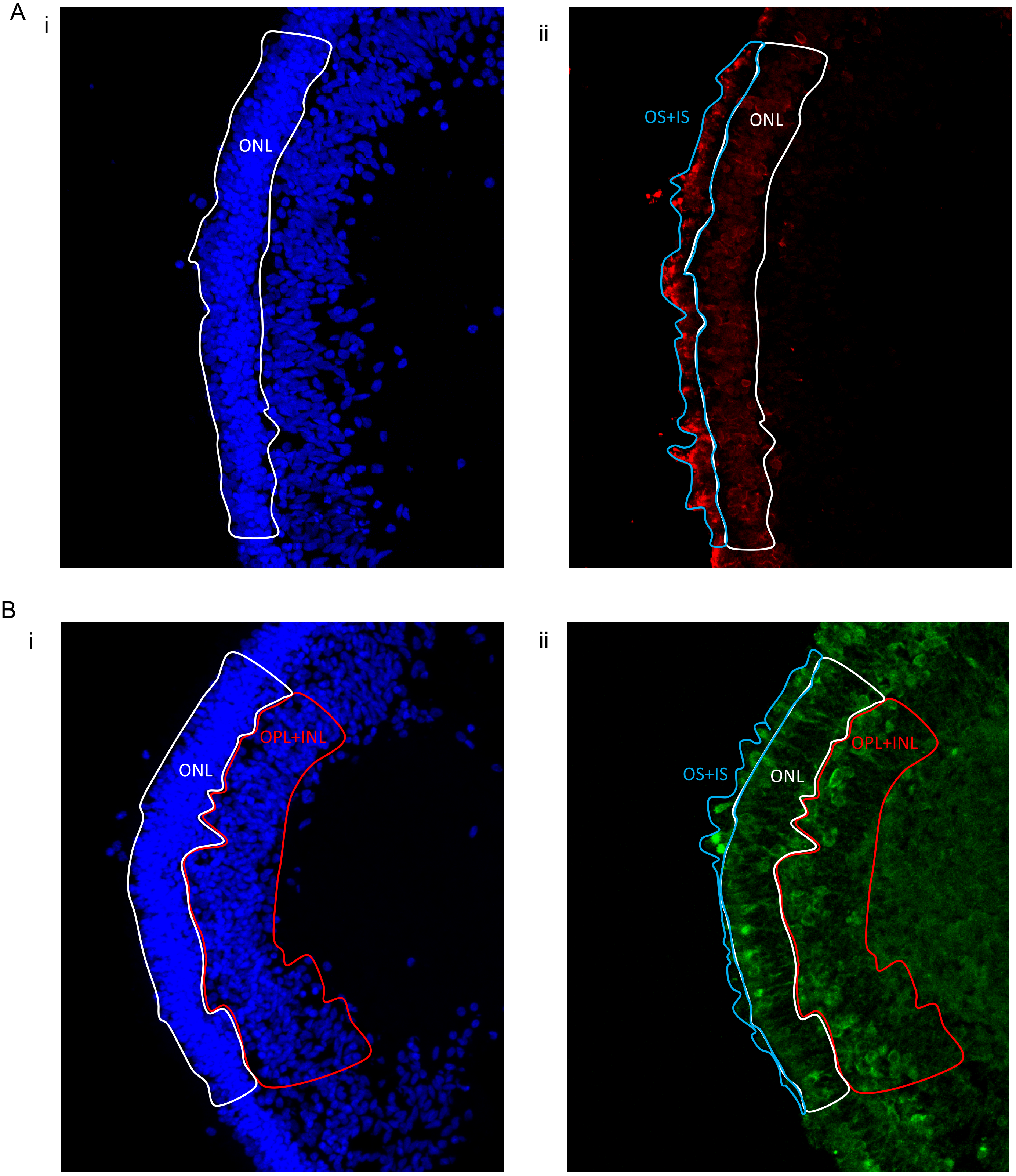

Supplementary Figure S6. Definition of regions for rhodopsin and L/M opsin TF ratio analysis in iPSC-ROs. *Regions of the iPSC-ROs with distinct ONL and INL were used for TF analysis.* ***A) (ii)*** *The borders of the ONL (white outline) were defined against DAPI staining (blue).* ***(ii)*** *The ONL outline was exported against the channel of interest, rhodopsin (red), and the OS+IS region (blue outline) was defined in relation to the defined ONL boundaries. The ONL TF (white outline) was measured and compared to the IS+OS TF (blue outline).* ***B) (i)*** *The ONL region (white outline) and the OPL+INL (red outline) region were defined against the DAPI channel (blue).* ***(ii)*** *The defined boundaries were exported against the channel of interest, LM opsin (green), and the OS+IS region was defined by using the ONL boundary as a guide. The ONL TF was measured in combination with the IS+OS region TF, and compared to the OPL+INL TF.*

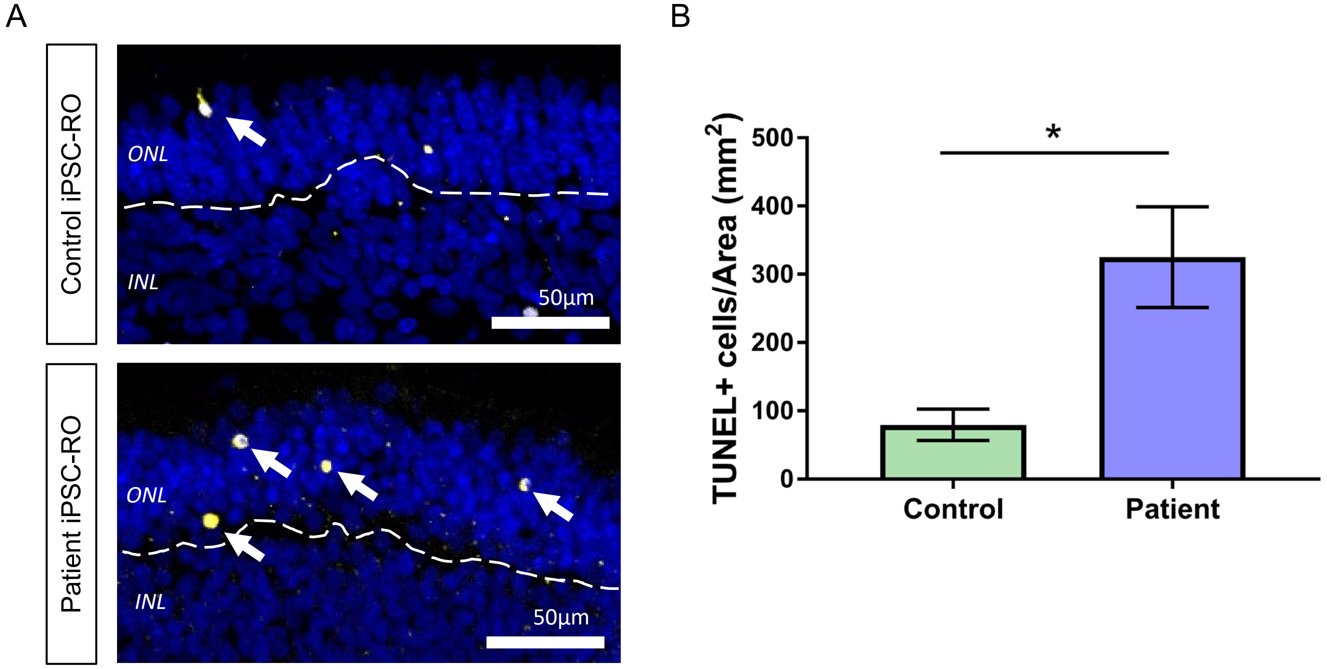

Supplementary Figure S7. Example of TUNEL assay in iPSC-ROs. A) *TUNEL+ staining (yellow) that colocalised (white arrows) with nuclei (blue) in the ONL of the iPSC-RO were counted and found to be increased in patient iPSC-RO ONL compared to control. The boundary between ONL and INL is indicated by the white dotted line.* ***B)*** *TUNEL assay in control and patient iPSC-ROs showed a higher number of TUNEL+ staining seen in the ONL of patient iPSC-ROs (unpaired t-test, *P<0.05, SEM from n=4-6 independent iPSC-ROs). (Control = Control 1, Patient = Patient 1 and 2).*
